# Early menarche and breast cancer risk: a systematic review and meta-analysis of 74 case–control studies

**DOI:** 10.1007/s10552-025-02096-y

**Published:** 2026-01-17

**Authors:** Fui-Ling Voon, Edmund Ui-Hang Sim

**Affiliations:** Faculty of Resource Science and Technology, University Malaysia Sarawak, 94300 Kota Samarahan, Sarawak Malaysia

**Keywords:** Menarche age, Early puberty, Female adolescent, Epidemiology, Breast neoplasms, Cancer risk

## Abstract

**Purpose:**

Early menarche is a known risk factor for breast cancer, as established by the Collaborative Group on Hormonal Factors in Breast Cancer in a 2012 reanalysis of data from forty-seven global epidemiological studies. Given recent changes in the average age at menarche, it is important to re-evaluate this association in the context of the past decade. This meta-analysis aimed to examine the relationship between early menarche, particularly before age 13, and female breast cancer risk by analyzing recently published observational studies and exploring this relationship across different regions of the world.

**Methods:**

Case–control studies published between January 2014 and February 2025 were systematically searched in PubMed, ScienceDirect, Scopus, and Google Scholar. Early menarche was defined as < 13 years. Pooled odds ratios (ORs) with 95% confidence intervals (CIs) were calculated using common-effect models when heterogeneity was low (*I*^2^ ≤ 30%) and random-effects models when heterogeneity was significant (*I*^2^ > 30%). Publication bias was assessed using Egger’s test.

**Results:**

Overall, results of the present meta-analyses show an increased association between menarche age < 13 and the risk of female breast cancer (OR = 1.15 [95% CI: 1.08 – 1.24]). Also, an increased association with breast cancer risk was found in the subgroup of menarche age < 12 (OR = 1.27 [95% CI: 1.09 – 1.48]). Lower OR was found in subgroup of menarche age ≥ 13 (OR = 0.89 [95% CI: 0.85 – 0.94]). For the geographical region subgroups, increased associations between menarche age < 13 and risk of breast cancer were found in Europe (OR = 1.15 [95% CI: 1.07 – 1.25]), North America (OR = 1.07 [95% CI: 1.03 – 1.11]), Oceania (OR = 1.15 [95% CI: 1.02 – 1.30]), and West Asia (OR = 1.70 [95% CI: 1.15 – 2.52]). Both population-based and hospital-based study designs demonstrated higher ORs for the association between menarche age < 13 and the risk of female breast cancer.

**Conclusions:**

This meta-analysis found a modest but statistically significant association between menarche before age 13 and increased breast cancer risk worldwide, while women who experienced menarche at age 13 or older had a lower risk. The association varied by region, with relatively higher odds observed in West Asia, Europe, Oceania, and North America. These results indicate an association rather than causation and are limited by the observational nature of the included studies.

**Supplementary Information:**

The online version contains supplementary material available at 10.1007/s10552-025-02096-y.

## Introduction

Breast cancer is the most prevalent cancer among women globally. It is a highly diverse cancer, encompassing several distinct subtypes that are categorized into four groups based on hormone receptors, which include estrogen receptor-positive (ER +), progesterone receptor-positive (PR +), human epidermal growth factor receptor 2-positive (HER2 +), and triple-negative breast cancer (TNBC) [1]. Breast cancer is caused by multiple factors [2], as its incidence, mortality, and survival rates differ significantly across regions, potentially due to variations in population demographics, lifestyle, genetic predispositions, and environmental conditions [3]. While screening can help alleviate the impact of breast cancer, it comes with drawbacks such as side effects, over-diagnosis, and higher costs. One approach to developing safer and more cost-effective targeted screening programs is by categorizing women based on their breast cancer risk factors [4]. Among the common risk factors for breast cancer, such as breast cancer gene 1 (*BRCA1*) and *BRCA2* gene mutations [5], older age [6], family history [7], dense breast tissue [8], and so on, an early onset of menarche, or puberty, is considered to be associated with a higher risk of breast cancer due to the increased number of ovulatory cycles over a lifetime, which results in greater exposure to ovarian hormones and thus a higher risk of breast cancer [9, 10].

Menarche is defined as the initial occurrence of menstruation in a female adolescent. According to research conducted in 67 countries and published between the 1960s and 1990s, the mean age at menarche was determined to be 13.53 years, with a standard deviation of ± 0.98 years [11]. In more recent years, menarche manifests between the ages of 10 and 16, with an average onset age of 12.4 years [12]. Menarche signifies the beginning of fertility, while menstruation, occurring roughly every 28 days (ranging from 21 to 45 days) with a mean interval of 32.2 days in the first gynaecologic year, involves the monthly shedding of the uterine endometrial lining, typically lasting three to seven days, with durations over ten days deemed abnormal [13]. Earlier onset of menarche is associated with stressful family environments, foster care, living with a stepparent, urban upbringing, and high socioeconomic status [14]. Dietary factors such as higher animal protein and lower vegetable protein intake [15], as well as the consumption of sugar-sweetened beverages [16], also contribute to earlier menarche. Additionally, being overweight or obese [17], and formula feeding during infancy [18], have been linked to an earlier onset of menarche.

The potential connection between early menarche and increased breast cancer risk has been extensively studied. The Gail model, presented in a 1989 report from the Breast Cancer Detection and Demonstration Project, highlighted the age of menarche as a significant risk factor for breast cancer. According to this model, women who experienced menarche before the age of 12 had a relative risk of 1.21 compared to those who had menarche after age 14 [19]. Nevertheless, a 2009 report from the Nurses’ Health Study, which mitigated the potential bias of enrolling higher-risk women, identified a smaller increase in relative risk of 1.10 for women who experienced menarche before age 12 [20]. A Moroccan case–control study demonstrated a significant association between early menarche (≤ 13 years) and an increased risk of breast cancer [21]. Additionally, a study found that tall women who experienced early menarche (≤ 13 years) had approximately twice the risk of developing estrogen/progesterone-positive (ER + PR +) tumors [22]. Conversely, a cohort study in the United Kingdom observed a lower risk in women whose menarche occurred at 15 years or older compared to those who began menstruating at ages 13–14 [23]. Interestingly, in this cohort study, menarche at 12 years or younger did not show a correlation with increased breast cancer risk [23].

The present meta-analysis investigates the association between age at menarche and the risk of female breast cancer, with particular focus on menarche occurring before age 13. This cut-off was selected based on a review of the literature and examination of data distributions across various populations [11, 24]. Using a PICO framework, the Population consisted of female breast cancer cases and controls from observational studies, the Intervention/Exposure was early menarche (< 13 years), the Comparison was later menarche (≥ 13 years), and the Outcome was breast cancer risk expressed as pooled odds ratios. Guided by this framework, the review addresses one primary question: *Does early menarche, compared with later menarche, increase the risk of developing female breast cancer?* By summarizing epidemiological evidence published in the last decade, this study aims to provide updated insights that may strengthen current risk assessment models and inform public health strategies, including those targeting childhood nutrition and body weight management.

## Methods

### Strategy of literature search

The present study adhered to the Preferred Reporting Items for Systematic Reviews and Meta-Analyses (PRISMA) guidelines [25]. Peer-reviewed papers were systematically searched across four online databases: ScienceDirect, PubMed, Scopus, and Google Scholar. Relevant articles were identified from the establishment of each database until 28 February 2025. Medical Subject Headings (MeSH) terms and keywords used in the search included (‘early menarche’ or ‘early puberty’) AND (‘breast cancer’ or ‘breast neoplasms’) AND (‘case–control’ or ‘cohort’ or ‘observational study’), applied to the titles and abstracts of the articles.

The full texts of suitable studies were downloaded for further screening. Duplicate copies were removed, and eligible studies were examined based on the inclusion and exclusion criteria. Additionally, backward citation tracking was conducted to ensure all relevant studies were included [26]. Two authors (FLV and EUHS) independently reviewed the full text of all potentially relevant citations (*n* = 600). Any disagreements during the screening process were resolved through consensus. After selection, both authors independently reviewed the included articles to extract and tabulate data in spreadsheets, with disagreements again resolved by consensus.

A protocol for this systematic review and meta-analysis was not prospectively registered in PROSPERO or any other protocol registry. However, all methodological steps, which include the search strategy, inclusion and exclusion criteria, and statistical analysis plan, were pre-specified and followed consistently throughout the study.

### Selection criteria

All articles identified through the literature search were systematically screened for relevance. Titles and abstracts were initially reviewed against predefined inclusion and exclusion criteria, followed by a full-text assessment of potentially eligible studies.

#### Inclusion criteria

Studies were included if they met the following criteria: (1) observational studies that included female breast cancer case and control populations; (2) reported data on menarche age categorized as < 13 years (e.g., < 13, 11–12, < 11, etc.); and (3) published as original research articles between 1 January 2014 and 28 February 2025; and (4) written in English.

#### Exclusion criteria

Studies were excluded if they met any of the following criteria: (1) review articles, meta-analyses, or letters to the editor; (2) did not provide data on menarche age < 13 years; (3) published in languages other than English; (4) contained duplicated or overlapping datasets; and (5) lacked sufficient data to perform statistical analyses.

Only studies published between 1 January 2014 and 28 February 2025 were included. This restriction was applied to capture recent epidemiological data that reflect contemporary trends in childhood nutrition, adiposity, environmental exposures, and age at menarche. Older studies were excluded to avoid mixing earlier birth cohorts whose risk profiles and menarche timing may not represent current populations.

Although cohort studies and clinical trials were retrieved during the screening process, they were excluded because they did not provide extractable raw case–control counts for early versus later menarche. The present meta-analysis required raw 2 × 2 data to calculate crude odds ratios consistently across all studies using the Mantel–Haenszel method. Cohort studies generally report adjusted hazard ratios (HR) or relative risks (RR) without presenting underlying raw counts, which prevented their inclusion without additional statistical assumptions or conversions. Clinical trials were likewise screened and excluded, as none provided case–control comparisons relevant to menarche age.

### Data extraction

In this review, “early menarche” was defined as menarche occurring before age 13 (e.g., ≤ 12, < 13, or similar thresholds depending on study categorization). This cut-off was selected based on epidemiological evidence indicating that the global mean age at menarche is approximately 12.4–13.5 years [11, 12], and because many large-scale breast cancer studies and prior meta-analyses apply < 13 years as the definition of early menarche. Although individual studies used different categorical ranges (e.g., ≤ 11, 11–12, ≤ 12), all were standardized into early (< 13) and later (≥ 13) menarche categories for pooled analysis. For consistency, participants with menarche at exactly 12 years were included in the early menarche group, and no participants were excluded on this basis. Age at menarche in the included studies was based on self-reported age at first menstruation, as recorded in each original article, which is consistent with standard epidemiological practice in reproductive health research.

After the initial selection of studies based on the inclusion and exclusion criteria, relevant data were extracted for analysis. The data extracted from every selected study included the following: author, year and country of publication, ethnicity, breast cancer subtypes, study design, sample size for case and control, source of subjects, matching criteria for case and control, and menarche age. For consistency and comparability across studies, raw case and control counts (i.e., the number of cases and controls with and without early menarche) were used as the primary data source. This approach was chosen because raw counts allow for a standardized calculation of crude odds ratios (ORs) across studies, ensuring uniformity in effect size estimation. By using raw counts, we minimize the heterogeneity introduced by different reporting formats (such as adjusted odds ratios or hazard ratios), allowing for a more consistent pooling of data in the meta-analysis. The use of raw counts ensures that the analysis is based on the most fundamental data provided in the studies, thereby reducing potential biases associated with adjustments for confounders that vary across studies. This method also avoids complications arising from the use of different statistical models or transformation of effect sizes. Additionally, the methodological quality of each study was assessed using the Newcastle–Ottawa Scale (NOS) [27], and the NOS scores were recorded.

### Statistical analysis

The data collected in this meta-analysis were the total number of participants and the number of participants who experienced menarche at the age < 13 in both case and control groups. The overall data were first analyzed as the main global result, and they were then further analyzed in four subgroups: (1) menarche age at < 12 and ≥ 13 years old, (2) study location or geographic region for menarche age at < 13, and (3) source of control, whether population- or hospital-based, for menarche age < 13.

For regional subgroup analyses, studies were categorized by geographic region based on the World Health Organization (WHO) and United Nations (UN) regional classification systems. Countries were grouped as (1) Europe: Italy, France, Germany, Norway, Sweden, Poland, United Kingdom, Spain, Finland; (2) North America: United States, Canada, Mexico; (3) South America: Brazil, Uruguay, Argentina; (4) West Asia: Iran, Saudi Arabia; (5) East Asia: China, Japan; (6) South Asia: India, Pakistan; (7) Southeast Asia: Malaysia, Thailand, Indonesia; (8) Oceania: Australia; (9) Africa: Central African Republic, Morocco, Nigeria. Geographic region was defined by the country where each study was conducted. In multi-country studies, the region of the primary recruitment site or majority population was used.

Dichotomous data were analyzed using the Mantel–Haenszel method and the inverse variance method, yielding odds ratio (OR) with respective 95% CIs and weight for each estimate, and the results were presented in forest plots. The pooled OR was symbolized by a solid diamond at the bottom of each forest plot. Statistical heterogeneity of the studies was assessed using the restricted maximum likelihood (REML) method and Q‐profile method, and the result was expressed as *τ*^2^ and *I*^2^ with 95% confidence intervals (CIs), as well as *p*-value. A common-effect (fixed-effect) model was used when heterogeneity was low (*I*^2^ ≤ 30%). When heterogeneity was greater than this threshold (*I*^2^ > 30%), a random-effects model was applied. When *I*^2^ > 50% or *p* < 0.10, heterogeneity was considered significant for pooled odds ratios. Egger’s test was applied to assess publication bias via a funnel plot asymmetry [28]. The result of *p* < 0.05 was considered as an indicator for the potential presence of publication bias. All meta-analyses were performed using R version 4.4.2 [29]. R statistics packages used for data analysis were ‘meta’ version 6.5.0 [30] and ‘metafor’ version 4.4.0 [31].

## Results

### Characteristics of included studies

**Fig. 1:**
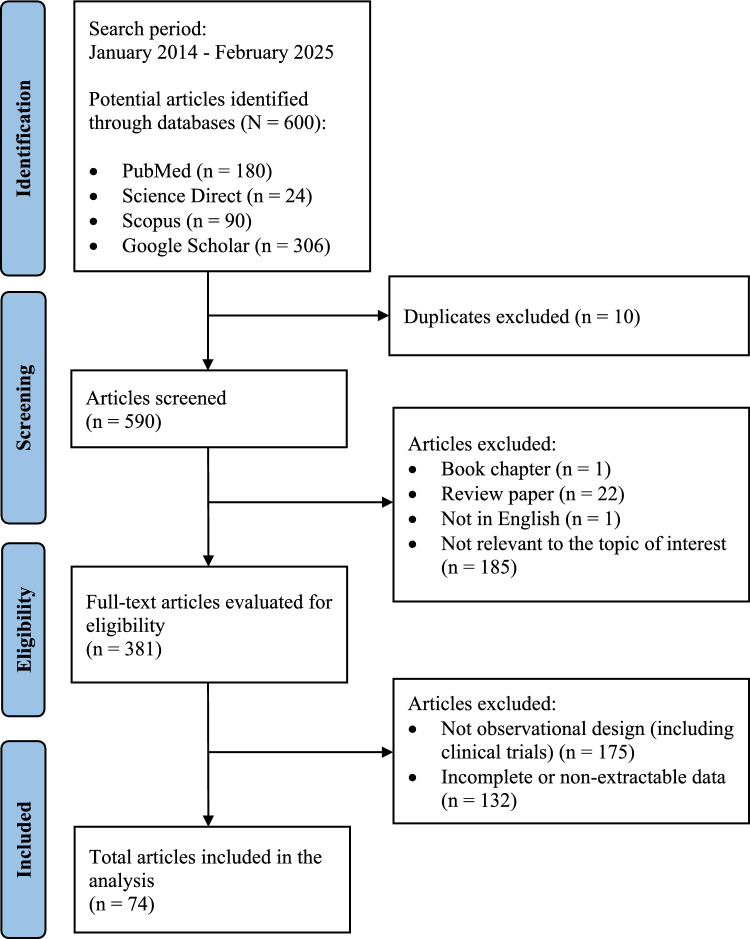
The Preferred Reporting Items for Systematic Reviews and Meta-Analyses (PRISMA) flow chart of 74 included studies in the meta-analysis

Figure 1 illustrates the study selection flowchart for this meta-analysis. Initially, 600 studies were retrieved from the literature search. After identifying and removing 10 duplicate studies, 209 articles were excluded due to being book chapters, review papers, in a foreign language (Chinese), or irrelevant. This left 381 full-text articles for eligibility screening. Among these, 175 were excluded for not being observational studies, and 132 were removed due to incomplete data. Ultimately, 74 articles were included in the analysis, comprising 66,650 cases and 145,172 controls. These 74 studies [32–105] were published between 2014 and 2023. The main characteristics of the selected studies are presented in Supplementary Material 1. Across the 74 case–control studies analyzed, the age at menarche ranged from 12 to 15 + years, with a weighted average of 12.78 years among cases and 12.77 years among controls. Early menarche (before age 13) was observed in 32.27% of cases and 29.81% of controls. The majority of cases (58.16%) and controls (61.00%) experienced menarche between 13 and 14 years, while fewer cases (9.57%) and controls (9.19%) reported menarche at age 15 and above.

### Meta-analyses

**Fig. 2:**
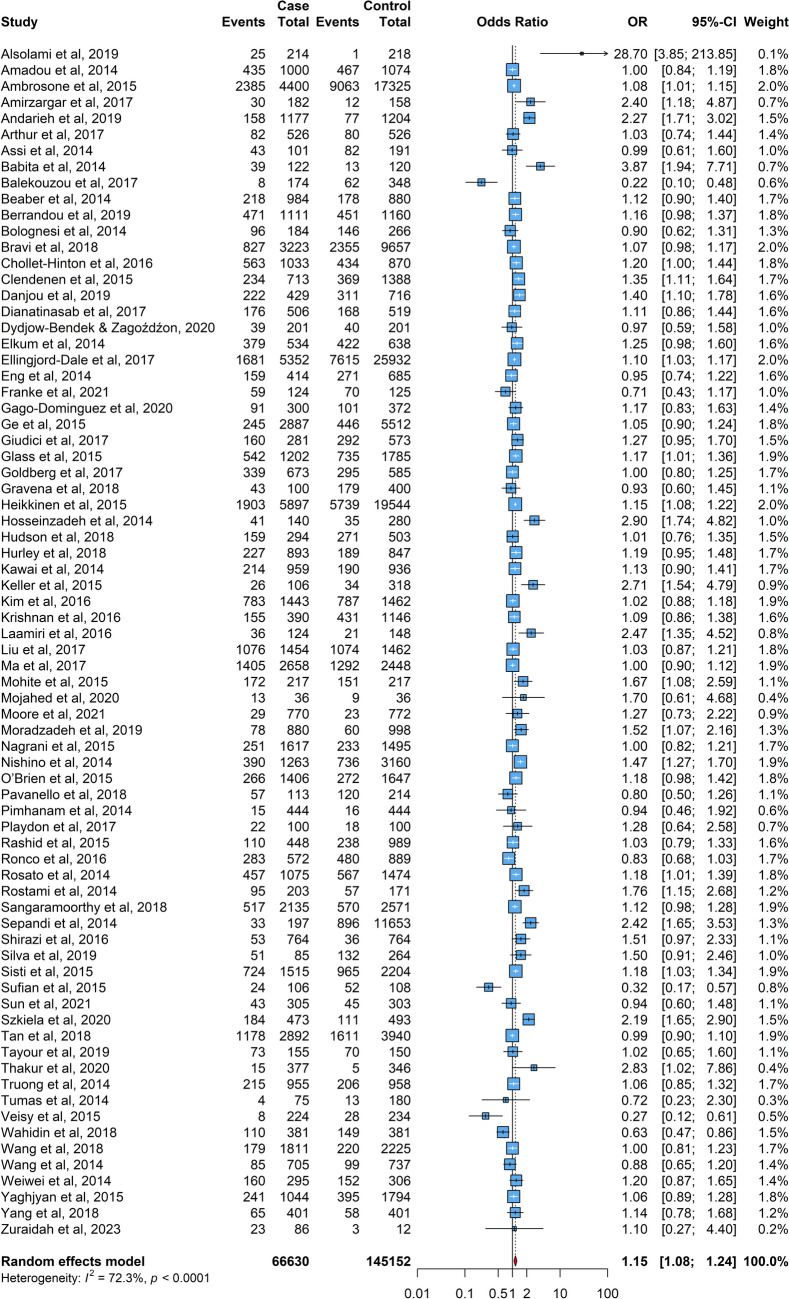
The forest plot depicting the pooled odds ratio (OR) from 74 studies worldwide, illustrating the association between menarche at age < 13 and the risk of female breast cancer. In this plot, “Case” represents the group of breast cancer patients, “Control” indicates the group of individuals without breast cancer, and “Events” signifies the number of cases or controls who experienced menarche before the age of 13

**Fig. 3:**
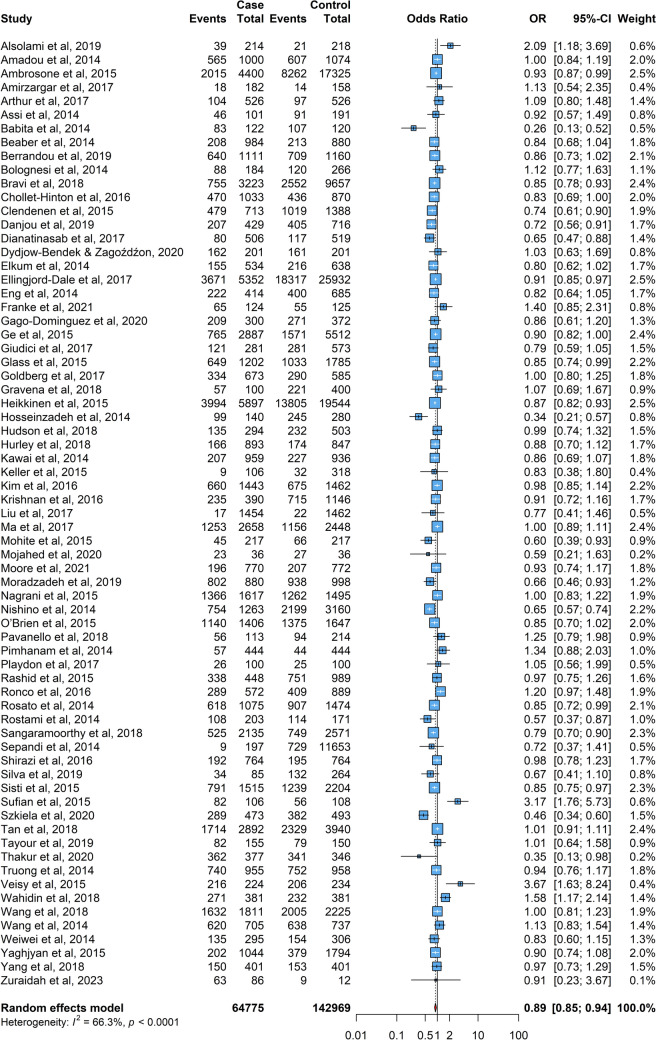
The forest plot displaying the pooled odds ratio (OR) for the association between menarche at age ≥ 13 and the risk of female breast cancer. In this plot, “Case” represents the group of breast cancer patients, “Control” refers to the group of individuals without breast cancer, and “Events” indicates the number of cases or controls who experienced menarche at age ≥ 13

**Fig. 4:**
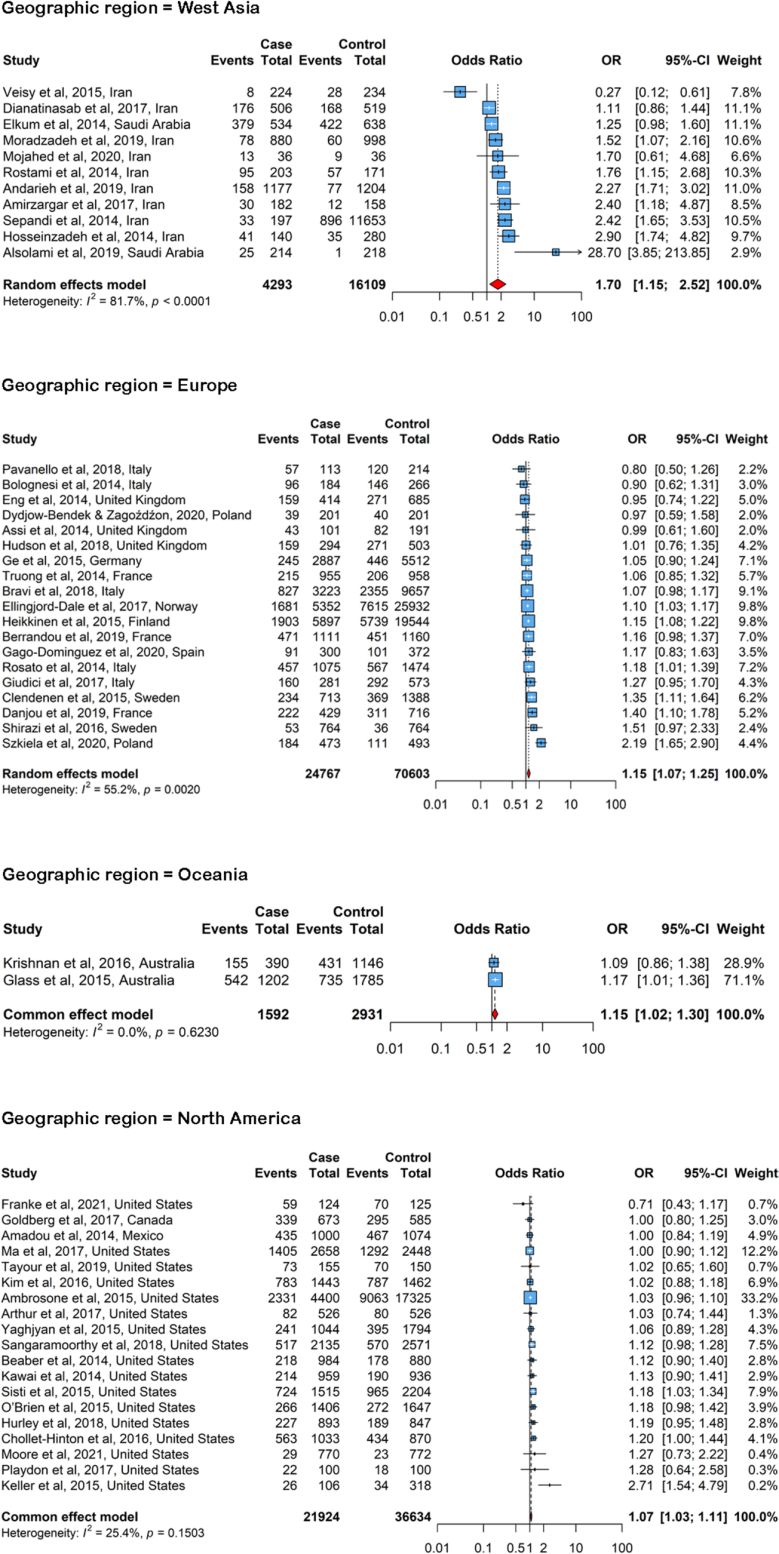
The forest plot illustrates the pooled odds ratio (OR) for the association between menarche before age 13 and the risk of female breast cancer across four global regions: West Asia, Europe, Oceania, and North America. In this plot, “Case” identifies individuals with breast cancer, “Control” represents those without breast cancer, and “Events” denotes the number of people in each group who had menarche before age 13

**Fig. 5:**
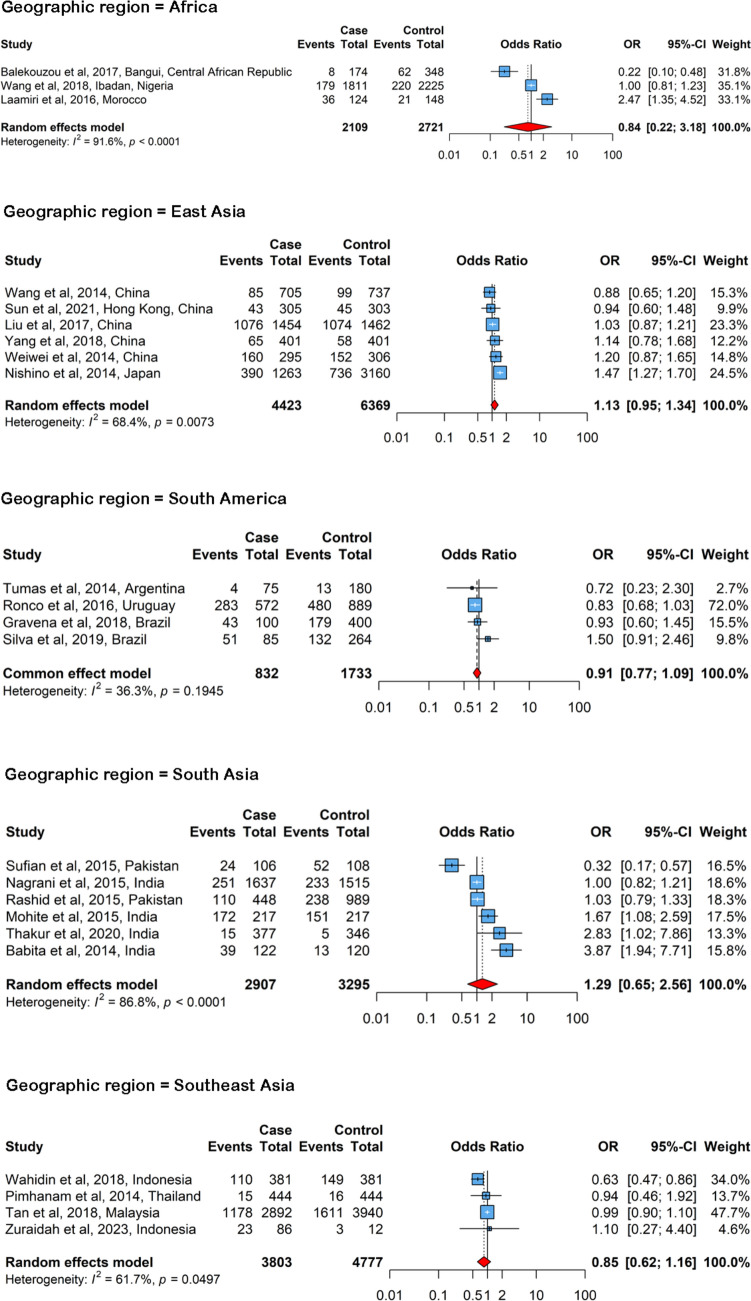
Forest plots showing regional associations between menarche before age 13 and breast cancer risk in Africa, East Asia, South America, South Asia, and Southeast Asia. The wider confidence intervals observed in these analyses indicate reduced statistical precision due to smaller sample sizes and fewer studies

**Fig. 6:**
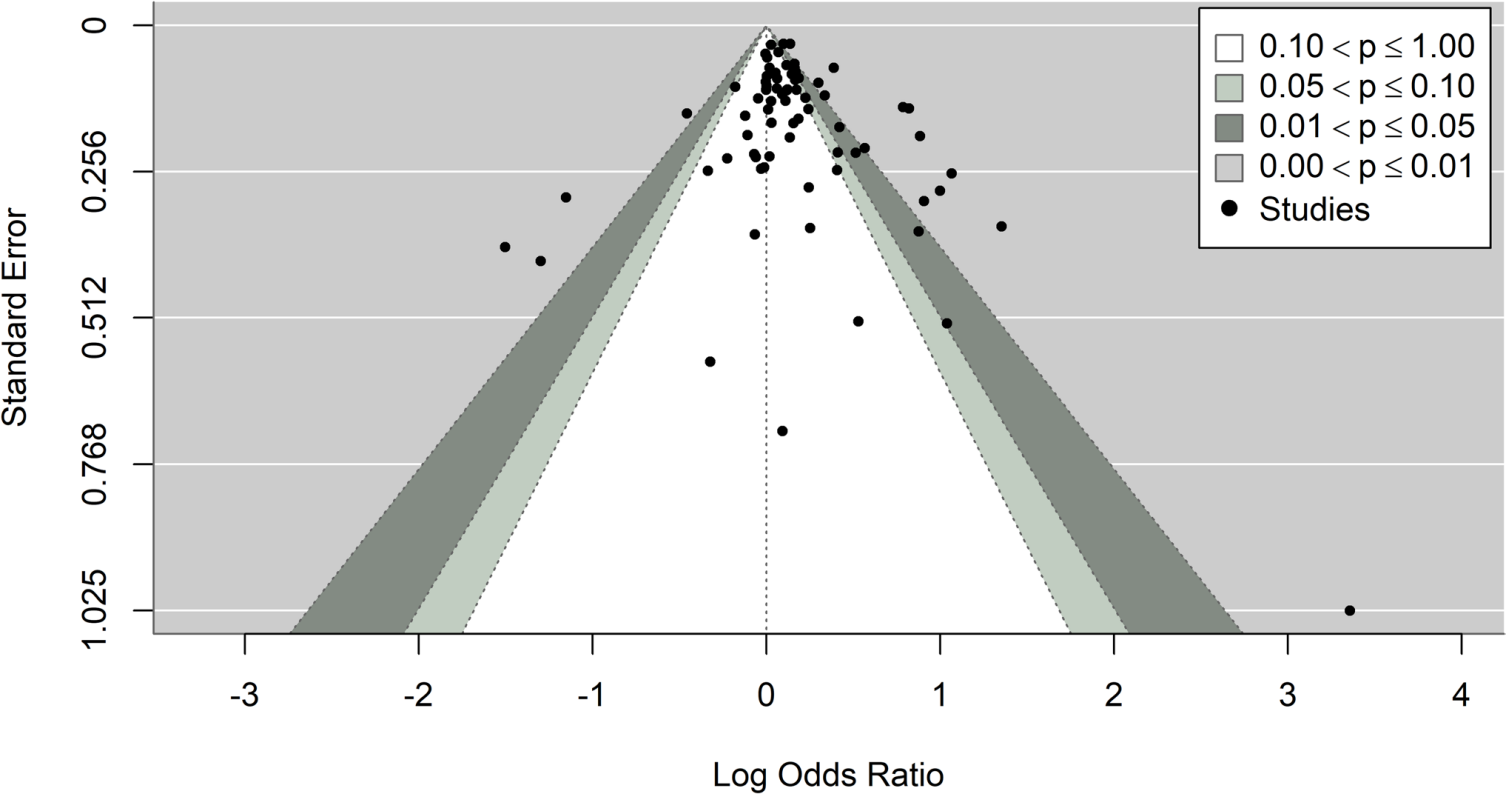
Contour-enhanced funnel plot of included studies. The plot is centered on a log odds ratio of zero (null effect), with dots representing the dispersion of effect sizes on the x-axis against the standard error on the y-axis. Different shading colors indicate different *p*-value ranges: Dots in the unshaded (white) region correspond to *p*-values > 0.1, the light-gray shaded region indicates *p*-values between 0.1 and 0.05, the dark-gray region indicates *p*-values between 0.05 and 0.01, and the region outside the funnel corresponds to *p*-values below 0.01

The results of each meta-analysis were presented in Figs. 2, 3, 4, 5, 6. The pooled OR for overall data from 74 studies was 1.15 (95% CI: 1.08–1.24) as portrayed in Fig. 2, indicating an association between menarche before age 13 and an increased risk of female cancer across the studies. Further analysis of the subgroup with menarche before age 12 yielded a pooled OR of 1.27 (95% CI: 1.09–1.48). For the subgroup with menarche at age 13 or older (Fig. 3), the pooled OR was 0.89 (95% CI: 0.85 – 0.94), suggesting a lower risk in this group.

In the subgroup analysis by region, the pooled ORs for Europe (OR = 1.15, 95% CI: 1.07–1.25), North America (OR = 1.07, 95% CI: 1.03–1.11), Oceania (OR = 1.15, 95% CI: 1.02–1.30), and West Asia (OR = 1.70, 95% CI: 1.15–2.52) were consistent with regional differences in the association between menarche age and breast cancer risk. These results are depicted in Fig. 4.

Associations that were not statistically significant were observed in Africa (OR = 0.84, 95% CI: 0.22 – 3.18), East Asia (OR = 1.13, 95% CI: 0.95 – 1.34), South America (OR = 0.91, 95% CI: 0.77 – 1.09), South Asia (OR = 1.29, 95% CI: 0.65 – 2.56), and Southeast Asia (OR = 0.85, 95% CI: 0.62 – 1.16). Although the overall direction of effect was generally consistent with the global trend, the magnitude of association was small and the precision of estimates varied across regions, reflecting the limited number of studies and wide confidence intervals in some subgroups. To improve interpretability, the results for South Asia and East Asia are presented separately, highlighting potential regional differences in sample size and population characteristics. The forest plots for these regions are shown in Fig. 5.

Subgroup analysis based on the source of subjects showed associations in both population-based and hospital-based study designs. In the population-based subgroup, the pooled OR was 1.11 (95% CI: 1.08 – 1.14), while the hospital-based subgroup had a pooled OR of 1.26 (95% CI: 1.06 – 1.51).

A summary of all analyses is available in Table 1, and the data extracted from the 74 studies can be found in Supplementary Material 2.

**Table 1:** Pooled odds ratio for different populations and ages of menarche in the meta-analyses

Population	Age of menarche (years old)	Number of studies	Statistical model	Pooled OR (95% CI)	*p*-value^a^	*p*-value^b^ (Publication bias)	*p*-value^c^ (Heterogeneity)	*I*^2^% (95% CI)	*τ*^2^ (95% CI)
All	< 13, < 12, < 10	74	Random-effects model	**1.153 (1.076–1.236)**	< 0.001*	0.171	< 0.001	72.3 (65.2–78.0)	0.06 (0.08–0.25)
All	< 12, < 10	26	Random-effects model	**1.267 (1.087–1.475)**	0.002*	0.051	< 0.001	74.1 (62.0–82.4)	0.11 (0.09–0.73)
All	≥ 13	69	Random-effects model	**0.894 (0.849–0.943)**	< 0.001*	0.975	< 0.001	66.3 (56.7–73.8)	0.03 (0.04–0.13)
Africa	< 13, < 12	3	Random-effects model	0.836 (0.220–3.184)	0.793	0.873	< 0.001	91.6 (78.4–96.7)	1.31 (0.28–58.38)
East Asia	< 13, < 12	6	Random-effects model	1.127 (0.946–1.343)	0.181	0.298	0.007	68.4 (25.4–86.6)	0.03 (0.00–0.19)
Europe	< 13, < 12	19	Random-effects model	**1.154 (1.068–1.246)**	< 0.001*	0.592	0.002	55.2 (25.0–73.3)	0.01 (0.00–0.08)
North America	< 13, < 12	19	Common-effect model	**1.069 (1.029–1.110)**	< 0.001*	0.112	0.150	25.4 (0.0–57.3)	0.00 (0.00–0.06)
Oceania	< 13	2	Common-effect model	**1.150 (1.015–1.304)**	0.028*	N/A	0.623	0.0 (0.0–0.0)	0.00 (0.00–0.00)
South America	< 13, < 12	4	Common-effect model	0.911 (0.765–1.085)	0.298	0.597	0.195	36.3 (0.0–77.9)	0.04 (0.00–1.26)
South Asia	< 13, < 12	6	Random-effects model	1.290 (0.651–2.557)	0.465	0.526	< 0.001	86.8 (73.5–93.4)	0.65 (0.20–4.67)
Southeast Asia	< 13, < 12	4	Random-effects model	0.849 (0.622–1.159)	0.301	0.573	0.050	61.7 (0.0–87.1)	0.05 (0.00–0.70)
West Asia	< 13, < 12, < 10	11	Random-effects model	**1.699 (1.145–2.520)**	0.008*	0.463	< 0.001	81.7 (68.4–89.4)	0.35 (0.17–3.05)
Population-based control	< 13, < 12	39	Random-effects model	**1.107 (1.080–1.135)**	< 0.001*	0.832	0.015	35.9 (5.1–56.6)	0.00 (0.00–0.06)
Hospital-based control	< 13, < 12, < 10	35	Random-effects model	**1.263 (1.058–1.507)**	0.010*	0.108	< 0.001	83.3 (77.6–87.5)	0.23 (0.17–0.70)

*OR* odds ratio, *CI* confidence interval, *N/A* Not available

^a^Random-effects model was used when the *p*-value for heterogeneity test was < 0.10 or *I*^2^ > 50%; otherwise, the common-effect model was used

^b^Egger’s test to evaluate publication bias, *p*-value of < 0.05 is considered statistically significant

^c^*p*-value of < 0.10 is considered statistically significant for Q statistics

*Significant *p*-value (< 0.05) for pooled OR

### Publication bias

The publication bias of the 74 included studies was evaluated with Egger’s test. The shape of the contour-enhanced funnel plot as depicted in Fig. 6 did not show any obvious asymmetry in the overall meta-analysis. Overall, there was no significant publication bias found among these 74 studies (*p* = 0.12). Moreover, there was no statistically significant publication bias found in any of the subgroup analyses. Summary of publication bias is available in Table 1.

## Discussion

The meta-analysis of 74 studies worldwide revealed an association between early menarche (before age 13) and an increased risk of female breast cancer, with a pooled odds ratio (OR) of 1.15, reflecting a 15% higher likelihood of developing breast cancer compared to those with later menarche. Although statistically significant, this effect size was modest. An odds ratio of approximately 1.15 indicates a small increase in risk and should be interpreted cautiously. Early menarche is better understood as a weak but consistent risk factor that contributes alongside multiple reproductive, genetic, lifestyle, and environmental influences. On its own, the magnitude is unlikely to have substantial clinical impact at the individual level and may partly reflect residual confounding and variability across study designs. Notably, the data for menarche before age 12 showed an even higher pooled OR of 1.27, indicating an increased risk, suggesting that earlier menarche may have a stronger impact on breast cancer development. On the contrary, menarche at age 13 or older was associated with a lower pooled OR of 0.89, indicating a reduced likelihood of developing breast cancer compared to those with earlier menarche. These results suggest a modest increase in breast cancer risk among females who experienced menarche at a younger age, likely due to the combined influence of factors such as prolonged estrogen exposure [106] across diverse populations. During puberty, hormones and growth factors, notably estrogen and insulin-like growth factor-1 (IGF-1), play a crucial role in breast development [107]. This period is marked by rapid cell proliferation, leading to ductal branching and the creation of terminal end buds [107, 108]. These buds later develop into terminal ductal lobular units, which are the primary sites where the majority of breast cancers begin [108, 109]. Therefore, early menarche likely increases the risk of breast cancer primarily due to these developmental processes [110].

The regions included in this meta-analysis exhibited varying associations between menarche before age 13 and the risk of breast cancer. The West Asia subgroup showed the strongest association (OR = 1.70), suggesting a higher risk of breast cancer development in this region, followed by Europe and Oceania (both OR = 1.15), and North America (OR = 1.07). It is important to note that regional variability may reflect differences in statistical power and precision rather than true biological differences. The pooled estimates for Africa, East Asia, South America, South Asia, and Southeast Asia were not statistically significant. The smaller effect sizes and wider confidence intervals in these regions suggest reduced statistical power, likely due to the limited number of available studies and smaller sample sizes. The lack of statistical significance should therefore be interpreted as a reflection of imprecision rather than absence of effect. In addition, publication year does not necessarily correspond to the period in which participants were born or when breast cancer cases were diagnosed. Secular trends in childhood nutrition, height growth, obesity, and age at menarche differ across birth cohorts and regions, and many of the included studies likely drew on women diagnosed decades earlier. Therefore, part of the regional variability may reflect differences in underlying birth cohorts rather than true geographic differences.

Moreover, differences in data quality, diagnostic period, and population characteristics may further contribute to regional variability. To capture this heterogeneity more clearly, the results for South Asia and East Asia are discussed separately, acknowledging their distinct epidemiological and cultural contexts. For instance, normal breast epithelium is more likely to be estrogen receptor-positive (ER +) in White Australian women than in Japanese women [111], which may be linked to variations in the structure and gene expression of normal breast tissue. Asian women in Asia generally exhibit lower breast cancer rates despite having smaller, denser breasts compared to Caucasian women. Nevertheless, within Asian populations, cancer risk may correlate with breast density [112]. Regional variability may also be influenced by differences in menopausal status and tumor receptor subtypes (such as ER/PR positivity). Ideally, the association between early menarche and breast cancer would be examined separately for premenopausal and postmenopausal women, as well as across hormone receptor subtypes. However, these variables were not consistently reported across studies, particularly in low- and middle-income regions, preventing meaningful stratification by menopausal or tumor biology subgroups. These differences highlight the importance of considering both the size and precision of regional effect estimates when interpreting global trends in early menarche and breast cancer risk.

On the other hand, a cross-sectional study comparing breast cancer risks among Lebanese and Lebanese American women revealed that Lebanese women had higher risk factors for developing breast cancer [113]. This study showed that Lebanese women had higher rates of smoking (both cigarettes and hookah) and tended to live closer to highways or main roads, whereas Lebanese American women breastfed for longer periods, exercised more, and consumed significantly more vegetables and fruits, all of which are protective against breast cancer [113]. Furthermore, a case–control study by Ziegler et al*.* (1993) investigated breast cancer risk factors among Asian American women versus women in Asia. The study found that Asian American women had higher breast cancer rates, largely due to Western lifestyle adoption. Key factors include dietary changes, reproductive behaviors, and overall lifestyle differences, indicating that environmental and lifestyle factors significantly contribute to breast cancer risk beyond genetic predispositions [114].

Subgroup analyses using both population-based and hospital-based controls showed an association between early menarche (before age 13) and the risk of female breast cancer, suggesting consistency in the findings across different control source types. Ideal control groups in epidemiologic studies are scarce, necessitating both theoretical and empirical efforts to minimize biases. Studies across various diseases, including vulvar cancer [115], idiopathic pulmonary fibrosis [116], and stroke [117], had identified a selection bias when hospital-based data are used in epidemiological or case–control studies, highlighting the value of population-based data for providing more accurate insights into patient characteristics. Hospital controls, suited for hospital cases, may not adequately represent the general population, whereas population controls are preferred despite their expense and procurement challenges [118]. Nonetheless, it has been suggested that carefully selected hospital controls can yield risk estimates comparable to those from population controls for a range of diseases [119].

It is important to note that there has been a secular decline in the age of menarche in many populations over the past century [120]. This trend may influence the relationship between age at menarche and breast cancer risk, with earlier menarche potentially conferring a higher risk. The current analysis indicates that women in regions such as West Asia, Europe, and North America, where secular trends have been most pronounced, may exhibit a heightened risk of breast cancer due to earlier menarche. Future research should consider how these trends in menarche timing, in conjunction with other environmental and lifestyle factors, influence breast cancer risk across generations. Regional differences in the age at menarche could be influenced by various factors, including socioeconomic conditions, cultural practices, and dietary patterns [120]. For instance, women in West Asia, Europe, and North America have generally experienced earlier menarche compared to populations in other regions, potentially contributing to the higher breast cancer risk observed in these areas. These findings emphasize the importance of developing region-specific risk assessment models to account for geographic variability in risk factors and outcomes.

An important consideration in this meta-analysis is the exclusion of cohort studies, which could offer valuable insights into the association between early menarche and breast cancer risk. Cohort studies, particularly prospective ones, avoid the differential recall bias inherent in case–control studies, where individuals with breast cancer may recall the timing of menarche differently compared to controls. This makes cohort studies less susceptible to recall bias [121], and their use could strengthen the evidence on this association. However, the inclusion criteria of this meta-analysis focused on observational studies that reported raw case and control counts, as this allowed for a more standardized and consistent approach to pooling data across studies. Cohort studies typically report results in the form of hazard ratios (HRs) or relative risks (RRs), which differ from the raw case–control counts required for the present meta-analysis. This data format difference posed a challenge to integrating cohort studies into the current analysis.

A literature search had been conducted and identified two relevant cohort studies. However, these studies reported their findings using different metrics (one with HRs [23] and the other with ORs [122] derived from adjusted models), which prevented their inclusion in the pooled analysis due to the absence of raw case–control data. Converting these results into a common metric would introduce additional assumptions and methodological complexities, potentially compromising the reliability of the estimates. Despite this, a discussion of these cohort studies has been included in this manuscript, acknowledging their findings and highlighting the complementary evidence they provide in relation to the case–control studies included in the present analysis. Future research may benefit from incorporating both cohort and case–control data, potentially pooling the results from cohort studies separately to avoid such discrepancies.

In addition to the findings from the case–control studies included in this meta-analysis, two cohort studies provide valuable insights into the relationship between menarche age and breast cancer risk. Yang et al. (2022) found that compared to menarche at age 13, menarche at age ≤ 11 years was significantly associated with an increased risk of early-onset breast cancer at age 40 (OR = 2.62, 95% CI: 1.38–4.97, *p* = 0.003), while menarche at age ≥ 16 years was significantly associated with a decreased risk (OR = 0.13, 95% CI: 0.03–0.53, *p* = 0.005) [122]. This suggests that earlier menarche may be a stronger predictor of early-onset breast cancer. In a similar vein, Bodicoat et al. (2014) reported that women whose menarche occurred at age 15 or older had a lower risk of breast cancer (OR = 0.78, 95% CI: 0.62–0.99) compared to those with menarche at age 13–14 [23]. However, they did not find an increased risk of breast cancer for women with menarche at age 12 or younger. These findings align with the results of our meta-analysis, further supporting the association between later menarche and a reduced risk of breast cancer, and suggesting that menarche at a younger age may not always be associated with higher risk, depending on the context.

The present meta-analysis faces several limitations, which include heterogeneity in study designs and populations, varying definitions and measurements across studies, and potential confounding factors such as family history and lifestyle variables that may not be consistently controlled. A key limitation of this meta-analysis is the reliance on raw numbers to calculate odds ratios, as this approach does not account for potential confounding variables. While adjusted odds ratios can provide more precise estimates by controlling for confounders, their inconsistent availability and the variability in adjustment models across studies precluded their use in this analysis. Despite this limitation, the use of raw data ensures methodological consistency and minimizes the heterogeneity that could arise from combining studies with differing adjustment factors. Also, limiting inclusion to studies from the past 10 years improves relevance to current populations but may have excluded older research with valuable longitudinal insight. Nonetheless, secular trends in age at menarche and breast cancer risk factors justify prioritizing recent data.

Additionally, the inability to obtain and analyze the data for a full range of specific menarche ages due to the varying categorization of menarche ages in the selected articles poses a significant constraint. Menopausal status and ER/PR receptor subtype were inconsistently reported across the included case–control studies, and were often missing entirely in studies from low- and middle-income regions. Consequently, it was not possible to assess whether regional differences in the association between early menarche and breast cancer risk were influenced by menopausal distribution or hormone-receptor-positive disease, nor to compare associations separately for premenopausal and postmenopausal women. On top of that, some regional subgroup estimates were based on a small number of studies or participants, which reduced statistical power and resulted in wider confidence intervals, limiting precision in regional comparisons. As most studies did not report birth cohort or year of diagnosis, we could not adjust for temporal changes in height growth, weight gain, or secular declines in menarche age, which may partly explain regional variability. Also, retrospective case–control designs introduce risks of recall and selection biases, and variability in risk estimates between studies, as well as differences in data quality and reporting, further challenge the generalizability of findings.

Furthermore, differences in study design, quality, and sources of bias may contribute to the observed heterogeneity in results. Population-based studies generally provide more representative data, whereas hospital-based studies may introduce selection biases, potentially affecting the magnitude and precision of the observed associations. The quality of the included studies varied, with lower-quality studies possibly yielding less reliable estimates. Additionally, publication bias could have inflated the estimates, as studies with significant findings are more likely to be published. Regional variations may reflect differences in population characteristics, healthcare systems, and environmental exposures, all of which could influence both the risk of breast cancer and the age at menarche. These limitations emphasize the need for cautious interpretation and highlight the areas for future research refinement.

Taken together, the results of this meta-analysis suggest that early menarche is associated with a modestly higher risk of breast cancer. However, the magnitude of this effect is small and should be interpreted with caution. As all included studies were observational, these findings demonstrate association but not causation. Moreover, the heterogeneity among regions may reflect differences in study design, diagnostic periods, and reporting practices rather than true biological variation. The reliance on case–control data introduces potential recall and selection biases, and the exclusion of cohort studies due to limited source of studies and differing data formats may have limited temporal inference. Additionally, incomplete information on menopausal status and hormone receptor subtypes across studies may have influenced pooled estimates. Future research integrating cohort data and individual participant-level analyses would strengthen causal interpretation and regional comparability.

## Conclusions

In conclusion, this meta-analysis of 74 case–control studies from 28 countries indicates that women who experienced menarche before age 13 have a modestly increased risk of breast cancer (OR = 1.15), while those with menarche at age 13 or older show a correspondingly lower risk. Regional differences were observed, with higher odds in West Asia, Europe, Oceania, and North America. Nevertheless, these patterns may partly reflect variations in study availability, population characteristics, and diagnostic timing. The findings provide updated evidence supporting early menarche as a long-recognized reproductive risk factor for breast cancer, yet the small effect size indicates that it is one of many contributors to overall risk. Given the observational nature of the included studies, these results should not be interpreted as causal. Several limitations, including the reliance on case–control data and incomplete reporting of menopausal and receptor status, necessitate cautious interpretation of the findings. Future studies integrating cohort data and region-specific analyses may help clarify the influence of menarche timing within evolving population contexts.

## Supplementary Information

Below is the link to the electronic supplementary material.

Supplementary file1 (DOCX 48 KB)

Supplementary file2 (CSV 7 KB)

## Data Availability

All data generated or analysed during this study were included in the previously referenced published articles.

## Abbreviations

BRCA1: Breast cancer gene 1
BRCA2: Breast cancer gene 2
CI: Confidence interval
ER: Estrogen receptor
ER +: Estrogen receptor-positive (ER +)
HER2: Human epidermal growth factor receptor 2
HR: Hazard ratio
IGF-1: Insulin-like growth factor-1
N/A: Not available
NOS: Newcastle–Ottawa Scale
OR: Odds ratio
PR: Progesterone receptor
PR +: Progesterone receptor-positive
RR: Relative risk
PRISMA: Preferred reporting items for systematic reviews and meta-analyses
TNBC: Triple-negative breast cancer

## References

[1] Shaath H, Elango R, Alajez NM (2021) Molecular classification of breast cancer utilizing long non-coding RNA (lncRNA) transcriptomes identifies novel diagnostic lncRNA panel for triple-negative breast cancer. Cancers (Basel) 13:1–16. 10.3390/cancers13215350 10.3390/cancers13215350 PMC8582428 34771513

[2] Salamat F, Niakan B, Keshtkar A et al (2018) Subtypes of benign breast disease as a risk factor of breast cancer: a systematic review and meta analyses. Iran J Med Sci 43:355–364 30046203 PMC6055208

[3] Hortobagyi GN, de la Garza Salazar J, Pritchard K et al (2005) The global breast cancer burden: variations in epidemiology and survival. Clin Breast Cancer 6:391–401. 10.3816/CBC.2005.n.043 16381622 10.3816/cbc.2005.n.043

[4] Mavaddat N, Pharoah PDP, Michailidou K et al (2015) Prediction of breast cancer risk based on profiling with common genetic variants. J Natl Cancer Inst. 10.1093/jnci/djv036 25855707 10.1093/jnci/djv036 PMC4754625

[5] Antoniou A, Pharoah PDP, Narod S et al (2003) Average risks of breast and ovarian cancer associated with BRCA1 or BRCA2 mutations detected in case series unselected for family history: a combined analysis of 22 studies. Am J Hum Genet 72:1117–1130 12677558 10.1086/375033 PMC1180265

[6] Jenkins EO, Deal AM, Anders CK et al (2014) Age-specific changes in intrinsic breast cancer subtypes: a focus on older women. Oncologist 19:1076–1083. 10.1634/theoncologist.2014-0184 25142841 10.1634/theoncologist.2014-0184 PMC4200998

[7] Braithwaite D, Miglioretti DL, Zhu W et al (2018) Family history and breast cancer risk among older women in the breast cancer surveillance consortium cohort. JAMA Intern Med 178:494–501. 10.1001/jamainternmed.2017.8642 29435563 10.1001/jamainternmed.2017.8642 PMC5876845

[8] Schreer I (2009) Dense breast tissue as an important risk factor for breast cancer and implications for early detection. Breast Care 4:89–92. 10.1159/000211954 20847885 10.1159/000211954 PMC2931066

[9] Clavel-Chapelon F (2002) Cumulative number of menstrual cycles and breast cancer risk: results from the E3N cohort study of French women. Cancer Causes Control 13:831–838 12462548 10.1023/a:1020684821837 PMC2001234

[10] Kelsey JL, Gammon MD, John EM (1993) Reproductive factors and breast cancer. Epidemiol Rev 15:36–47 8405211 10.1093/oxfordjournals.epirev.a036115

[11] Thomas F, Renaud F, Benefice E et al (2001) International variability of ages at menarche and menopause: patterns and main determinants. Hum Biol 73:271–290 11446429 10.1353/hub.2001.0029

[12] Marques P, Madeira T, Gama A (2022) Menstrual cycle among adolescents: girls’ awareness and influence of age at menarche and overweight. Rev Paul Pediatr. 10.1590/1984-0462/2022/40/2020494 35019010 10.1590/1984-0462/2022/40/2020494 PMC8734600

[13] Finer LB, Philbin JM (2014) Trends in ages at key reproductive transitions in the United States, 1951–2010. Womens Health Issues. 10.1016/j.whi.2014.02.002 24721149 10.1016/j.whi.2014.02.002 PMC4011992

[14] Wronka I, Pawlińska-Chmara R (2005) Menarcheal age and socio-economic factors in Poland. Ann Hum Biol 32:630–638. 10.1080/03014460500204478 16316918 10.1080/03014460500204478

[15] Berkey CS, Gardner JD, Frazier AL, Colditz GA (2000) Relation of childhood diet and body size to menarche and adolescent growth in girls. Am J Epidemiol 152:446–452 10981459 10.1093/aje/152.5.446

[16] Carwile JL, CWillett W, Spiegelman D et al (2015) Sugar-sweetened beverage consumption and age at menarche in a prospective study of US girls. Hum Reprod 30:675–683. 10.1093/humrep/deu349 25628346 10.1093/humrep/deu349 PMC4325672

[17] Almuhlafi M, Jamilah KA, Almutairi AF, Salam M (2018) Relationship between early menarche, obesity, and disordered eating behaviors: a school-based cross-sectional survey in Northern Saudi Arabia. Diabetes Metab Syndr Obes 11:743–751. 10.2147/DMSO.S180697 30532574 10.2147/DMSO.S180697 PMC6244586

[18] Lee HS (2021) Why should we be concerned about early menarche? Clin Exp Pediatr 64:26–27. 10.3345/cep.2020.00521 32683812 10.3345/cep.2020.00521 PMC7806408

[19] Gail MH, Brinton LA, Byar DP et al (1989) Projecting individualized probabilities of developing breast cancer for white females who are being examined annually. J Natl Cancer Inst 81:1879–1886 2593165 10.1093/jnci/81.24.1879

[20] Lacey JV, Kreimer AR, Buys SS et al (2009) Breast cancer epidemiology according to recognized breast cancer risk factors in the prostate, lung, colorectal and ovarian (PLCO) cancer screening trial cohort. BMC Cancer 9:1–8. 10.1186/1471-2407-9-84 19292893 10.1186/1471-2407-9-84 PMC2670317

[21] Khalis M, Charbotel B, Chajès V et al (2018) Menstrual and reproductive factors and risk of breast cancer: a case-control study in the Fez region, Morocco. PLoS ONE. 10.1371/journal.pone.0191333 29338058 10.1371/journal.pone.0191333 PMC5770054

[22] Ritte R, Lukanova A, Tjønneland A et al (2013) Height, age at menarche and risk of hormone receptor-positive and -negative breast cancer: a cohort study. Int J Cancer 132:2619–2629. 10.1002/ijc.27913 23090881 10.1002/ijc.27913

[23] Bodicoat DH, Schoemaker MJ, Jones ME et al (2014) Timing of pubertal stages and breast cancer risk: the Breakthrough Generations Study. Breast Cancer Res. 10.1186/bcr3613 24495528 10.1186/bcr3613 PMC3978643

[24] Meher T, Sahoo H (2024) Secular trend in age at menarche among Indian women. Sci Rep. 10.1038/s41598-024-55657-7 38443461 10.1038/s41598-024-55657-7 PMC10914750

[25] Moher D, Shamseer L, Clarke M et al (2016) Preferred reporting items for systematic review and meta-analysis protocols (PRISMA-P) 2015 statement. Revista Espanola de Nutricion Humana y Dietetica 20:148–160. 10.1186/2046-4053-4-1

[26] Sim CC, Sim EUH (2020) Over-expression of cyclo-oxygenase-2 predicts poor survival of patients with nasopharyngeal carcinoma: a meta-analysis. J Laryngol Otol 134:338–343. 10.1017/S0022215120000614 32172705 10.1017/S0022215120000614

[27] Wells G, Shea B, O’Connell D, et al The Newcastle-Ottawa Scale (NOS) for assessing the quality of nonrandomised studies in meta-analyses. In: https://www.ohri.ca/programs/clinical_epidemiology/oxford.asp.

[28] Egger M, Smith GD, Schneider M, Minder C (1997) Bias in meta-analysis detected by a simple, graphical test. BMJ 315:629–634. 10.1136/bmj.315.7109.629 9310563 10.1136/bmj.315.7109.629 PMC2127453

[29] R Core Team (2023) R: A language and environment for statistical computing

[30] Balduzzi S, Rücker G, Schwarzer G (2019) How to perform a meta-analysis with R: a practical tutorial. Evid Based Ment Health 22:153–160 31563865 10.1136/ebmental-2019-300117 PMC10231495

[31] Viechtbauer W (2010) Conducting meta-analyses in R with the metafor package. J Stat Softw 36:1–48

[32] Alsolami FJ, Azzeh FS, Ghafouri KJ et al (2019) Determinants of breast cancer in Saudi women from Makkah region: a case-control study (breast cancer risk factors among Saudi women). BMC Public Health. 10.1186/s12889-019-7942-3 31752790 10.1186/s12889-019-7942-3 PMC6873398

[33] Amadou A, Torres Mejia G, Fagherazzi G et al (2014) Anthropometry, silhouette trajectory, and risk of breast cancer in Mexican women. Am J Prev Med. 10.1016/j.amepre.2013.10.024 24512931 10.1016/j.amepre.2013.10.024

[34] Ambrosone CB, Zirpoli G, Hong CC et al (2015) Important role of menarche in development of estrogen receptor-negative breast cancer in African American women. J Natl Cancer Inst. 10.1093/jnci/djv172 26085483 10.1093/jnci/djv172 PMC4836800

[35] Amirzargar A, Hamzavi N, Mahmoodi M et al (2017) Association study between single nucleotide polymorphisms of vascular endothelial growth factor and risk of breast cancer among Iranian population. Basic Clin Cancer Res 9:12–20

[36] Andarieh M, Delavar M, Moslemi D et al (2019) Infertility as a risk factor for breast cancer: results from a hospital-based case-control study. J Cancer Res Ther 15:976–980. 10.4103/jcrt.JCRT_905_16 31603097 10.4103/jcrt.JCRT_905_16

[37] Arthur R, Wang Y, Ye K et al (2017) Association between lifestyle, menstrual/reproductive history, and histological factors and risk of breast cancer in women biopsied for benign breast disease. Breast Cancer Res Treat 165:623–631. 10.1007/s10549-017-4347-9 28643020 10.1007/s10549-017-4347-9 PMC5886737

[38] Assi V, Massat NJ, Thomas S et al (2015) A case-control study to assess the impact of mammographic density on breast cancer risk in women aged 40-49 at intermediate familial risk. Int J Cancer 136:2378–2387. 10.1002/ijc.29275 25333209 10.1002/ijc.29275

[39] Babita R, Kumar N, Karwasra RK et al (2014) Reproductive risk factors associated with breast carcinoma in a tertiary care hospital of north India: a case-control study. Indian J Cancer 51:251–255. 10.4103/0019-509X.146759 25494116 10.4103/0019-509X.146759

[40] Balekouzou A, Yin P, Pamatika CM et al (2017) Reproductive risk factors associated with breast cancer in women in Bangui: a case-control study. BMC Womens Health. 10.1186/s12905-017-0368-0 28264686 10.1186/s12905-017-0368-0 PMC5340027

[41] Beaber EF, Malone KE, Tang MTC et al (2014) Oral contraceptives and breast cancer risk overall and by molecular subtype among young women. Cancer Epidemiol Biomarkers Prev 23:755–764. 10.1158/1055-9965.EPI-13-0944 24633144 10.1158/1055-9965.EPI-13-0944 PMC4032363

[42] Berrandou T, Mulot C, Cordina-Duverger E et al (2019) Association of breast cancer risk with polymorphisms in genes involved in the metabolism of xenobiotics and interaction with tobacco smoking: a gene-set analysis. Int J Cancer 144:1896–1908. 10.1002/ijc.31917 30303517 10.1002/ijc.31917

[43] Bolognesi C, Bruzzi P, Gismondi V et al (2014) Clinical application of micronucleus test: a case-control study on the prediction of breast cancer risk/susceptibility. PLoS ONE 9:e112354. 10.1371/journal.pone.0112354 25415331 10.1371/journal.pone.0112354 PMC4240584

[44] Bravi F, Decarli A, Russo AG (2018) Risk factors for breast cancer in a cohort of mammographic screening program: a nested case–control study within the FRiCaM study. Cancer Med 7:2145–2152. 10.1002/cam4.1427 29654663 10.1002/cam4.1427 PMC5943434

[45] Chollet-Hinton L, Anders CK, Tse CK et al (2016) Breast cancer biologic and etiologic heterogeneity by young age and menopausal status in the Carolina Breast Cancer Study: a case-control study. Breast Cancer Res. 10.1186/s13058-016-0736-y 27492244 10.1186/s13058-016-0736-y PMC4972943

[46] Clendenen TV, Ge W, Koenig KL et al (2015) Genetic polymorphisms in vitamin D metabolism and signaling genes and risk of breast cancer: a nested case-control study. PLoS ONE. 10.1371/journal.pone.0140478 26488576 10.1371/journal.pone.0140478 PMC4619526

[47] Danjou AMN, Coudon T, Praud D et al (2019) Long-term airborne dioxin exposure and breast cancer risk in a case-control study nested within the French E3N prospective cohort. Environ Int 124:236–248. 10.1016/j.envint.2019.01.001 30658268 10.1016/j.envint.2019.01.001

[48] Dianatinasab M, Fararouei M, Mohammadianpanah M et al (2017) Hair coloring, stress, and smoking increase the risk of breast cancer: a case-control study. Clin Breast Cancer 17:650–659. 10.1016/j.clbc.2017.04.012 28549689 10.1016/j.clbc.2017.04.012

[49] Dydjow-Bendek D, Zagoźdźon P (2020) Total dietary fats, fatty acids, and omega-3/omega-6 ratio as risk factors of breast cancer in the Polish population - a case–control study. In Vivo 34:423–431. 10.21873/invivo.11791 31882509 10.21873/invivo.11791 PMC6984116

[50] Elkum N, Al-Tweigeri T, Ajarim D et al (2014) Obesity is a significant risk factor for breast cancer in Arab women. BMC Cancer. 10.1186/1471-2407-14-788 25351244 10.1186/1471-2407-14-788 PMC4532295

[51] Ellingjord-Dale M, Vos L, Tretli S et al (2017) Parity, hormones and breast cancer subtypes - results from a large nested case–control study in a national screening program. Breast Cancer Res. 10.1186/s13058-016-0798-x 28114999 10.1186/s13058-016-0798-x PMC5259848

[52] Eng A, Gallant Z, Shepherd J et al (2014) Digital mammographic density and breast cancer risk: a case–control study of six alternative density assessment methods. Breast Cancer Res. 10.1186/s13058-014-0439-1 25239205 10.1186/s13058-014-0439-1 PMC4303120

[53] Franke AA, Li X, Shvetsov YB, Lai JF (2021) Pilot study on the urinary excretion of the glyphosate metabolite aminomethylphosphonic acid and breast cancer risk: the multiethnic cohort study. Environ Pollut. 10.1016/j.envpol.2021.116848 33714786 10.1016/j.envpol.2021.116848 PMC8044054

[54] Gago-Dominguez M, Matabuena M, Redondo CM et al (2020) Neutrophil to lymphocyte ratio and breast cancer risk: analysis by subtype and potential interactions. Sci Rep. 10.1038/s41598-020-70077-z 32764699 10.1038/s41598-020-70077-z PMC7413522

[55] Ge I, Rudolph A, Shivappa N et al (2015) Dietary inflammation potential and postmenopausal breast cancer risk in a German case–control study. Breast 24:491–496. 10.1016/j.breast.2015.04.012 25987487 10.1016/j.breast.2015.04.012

[56] Giudici F, Scaggiante B, Scomersi S et al (2017) Breastfeeding: a reproductive factor able to reduce the risk of luminal B breast cancer in premenopausal White women. Eur J Cancer Prev 26:217–224. 10.1097/CEJ.0000000000000220 26849393 10.1097/CEJ.0000000000000220

[57] Glass DC, Heyworth J, Thomson AK et al (2015) Occupational exposure to solvents and risk of breast cancer. Am J Ind Med 58:915–922. 10.1002/ajim.22478 26010434 10.1002/ajim.22478

[58] Goldberg MS, Labrèche F, Weichenthal S et al (2017) The association between the incidence of postmenopausal breast cancer and concentrations at street-level of nitrogen dioxide and ultrafine particles. Environ Res 158:7–15. 10.1016/j.envres.2017.05.038 28595043 10.1016/j.envres.2017.05.038

[59] Gravena AAF, Lopes TCR, de Demitto M, O, et al (2018) The obesity and the risk of breast cancer among pre and postmenopausal women. Asian Pacific J Cancer Prevention 19:2429–2436. 10.22034/APJCP.2018.19.9.2429 10.22034/APJCP.2018.19.9.2429 PMC6249449 30255696

[60] Heikkinen S, Koskenvuo M, Malila N et al (2016) Use of exogenous hormones and the risk of breast cancer: results from self-reported survey data with validity assessment. Cancer Causes Control 27:249–258. 10.1007/s10552-015-0702-5 26667320 10.1007/s10552-015-0702-5

[61] Hosseinzadeh M, Ziaei JE, Mahdavi N et al (2014) Risk factors for breast cancer in Iranian women: a hospital-based case-control study in Tabriz, Iran. J Breast Cancer 17:236–243. 10.4048/jbc.2014.17.3.236 25320621 10.4048/jbc.2014.17.3.236 PMC4197353

[62] Hudson S, Vik Hjerkind K, Vinnicombe S et al (2018) Adjusting for BMI in analyses of volumetric mammographic density and breast cancer risk. Breast Cancer Res. 10.1186/s13058-018-1078-8 30594212 10.1186/s13058-018-1078-8 PMC6311032

[63] Hurley S, Goldberg D, Wang M et al (2018) Breast cancer risk and serum levels of per- and poly-fluoroalkyl substances: a case-control study nested in the California Teachers Study. Environ Health. 10.1186/s12940-018-0426-6 30482205 10.1186/s12940-018-0426-6 PMC6260688

[64] Kawai M, Malone KE, Tang MTC, Li CI (2014) Active smoking and the risk of estrogen receptor-positive and triple-negative breast cancer among women ages 20 to 44 years. Cancer 120:1026–1034. 10.1002/cncr.28402 24515648 10.1002/cncr.28402 PMC4090108

[65] Keller BM, Chen J, Daye D et al (2015) Preliminary evaluation of the publicly available Laboratory for Breast Radiodensity Assessment (LIBRA) software tool: comparison of fully automated area and volumetric density measures in a case–control study with digital mammography. Breast Cancer Res. 10.1186/s13058-015-0626-8 26303303 10.1186/s13058-015-0626-8 PMC4549121

[66] Kim J, Mersereau JE, Khankari N et al (2016) Polycystic ovarian syndrome (PCOS), related symptoms/sequelae, and breast cancer risk in a population-based case–control study. Cancer Causes Control 27:403–414. 10.1007/s10552-016-0716-7 26797454 10.1007/s10552-016-0716-7 PMC4981498

[67] Krishnan K, Baglietto L, Apicella C et al (2016) Mammographic density and risk of breast cancer by mode of detection and tumor size: a case–control study. Breast Cancer Res. 10.1186/s13058-016-0722-4 27316945 10.1186/s13058-016-0722-4 PMC4912759

[68] Laamiri FZ, Hasswane N, Kerbach A et al (2016) Risk factors associated with a breast cancer in a population of Moroccan women whose age is less than 40 years: A case control study. Pan African Med J. 10.11604/pamj.2016.24.19.8784 10.11604/pamj.2016.24.19.8784 PMC4992386 27583083

[69] Liu L-Y, Wang F, Cui S-D et al (2017) A case-control study on risk factors of breast cancer in Han Chinese women. Oncotarget 8:97217–97230 29228605 10.18632/oncotarget.21743 PMC5722557

[70] Ma H, Ursin G, Xu X et al (2017) Reproductive factors and the risk of triple-negative breast cancer in white women and African-American women: a pooled analysis. Breast Cancer Res. 10.1186/s13058-016-0799-9 28086982 10.1186/s13058-016-0799-9 PMC5237290

[71] Mohite VR, Pratinidhi AK, Mohite RV (2015) Reproductive risk factors and breast cancer: a case control study from rural India. Bangladesh J Med Sci 14:258–264. 10.3329/bjms.v14i3.21865

[72] Mojahed FH, Aalami AH, Pouresmaeil V et al (2020) Clinical evaluation of the diagnostic role of microRNA-155 in breast cancer. Int J Genomics. 10.1155/2020/9514831 10.1155/2020/9514831 PMC7495225 32964011

[73] Moore SC, Mazzilli KM, Sampson JN et al (2021) A metabolomics analysis of postmenopausal breast cancer risk in the cancer prevention study II. Metabolites 11:1–13. 10.3390/metabo11020095 10.3390/metabo11020095 PMC7916573 33578791

[74] Moradzadeh R, Mansournia MA, Ghiasvand R et al (2019) Impact of age at menarche on breast cancer: The assessment of recall bias. Arch Iran Med 22:65–70 30980640

[75] Nagrani R, Mhatre S, Boffetta P et al (2016) Understanding rural–urban differences in risk factors for breast cancer in an Indian population. Cancer Causes Control 27:199–208. 10.1007/s10552-015-0697-y 26589416 10.1007/s10552-015-0697-y

[76] Nishino Y, Minami Y, Kawai M et al (2014) Cigarette smoking and breast cancer risk in relation to joint estrogen and progesterone receptor status: A case–control study in Japan. Springerplus 3:1–15. 10.1186/2193-1801-3-65 24516791 10.1186/2193-1801-3-65 PMC3918095

[77] O’Brien KM, Sun J, Sandler DP et al (2015) Risk factors for young-onset invasive and in situ breast cancer. Cancer Causes Control 26:1771–1778. 10.1007/s10552-015-0670-9 26407954 10.1007/s10552-015-0670-9 PMC5119634

[78] Pavanello S, Varesco L, Gismondi V et al (2018) Leucocytes telomere length and breast cancer risk/ susceptibility: a case–control study. PLoS ONE. 10.1371/journal.pone.0197522 29782524 10.1371/journal.pone.0197522 PMC5962062

[79] Pimhanam C, Sangrajrang S, Ekpanyaskul C (2014) Tobacco smoke exposure and breast cancer risk in Thai urban females. Asian Pac J Cancer Prev 15:7407–7411. 10.7314/APJCP.2014.15.17.7407 25227850 10.7314/apjcp.2014.15.17.7407

[80] Playdon MC, Ziegler RG, Sampson JN et al (2017) Nutritional metabolomics and breast cancer risk in a prospective study. Am J Clin Nutr 106:637–686. 10.3945/ajcn 28659298 10.3945/ajcn.116.150912 PMC5525118

[81] Rashid MU, Muzaffar M, Khan FA et al (2015) Association between the bsmi polymorphism in the Vitamin D receptor gene and breast cancer risk: results from a Pakistani case-control study. PLoS ONE. 10.1371/journal.pone.0141562 26517870 10.1371/journal.pone.0141562 PMC4627649

[82] Ronco AL, De Stefani E, Mendoza B et al (2016) Mate intake and risk of breast cancer in Uruguay: a case–control study. Asian Pac J Cancer Prev 17:1453–1461. 10.7314/APJCP.2016.17.3.1453 27039789 10.7314/apjcp.2016.17.3.1453

[83] Rosato V, Bosetti C, Negri E et al (2014) Reproductive and hormonal factors, family history, and breast cancer according to the hormonal receptor status. Eur J Cancer Prev 23:412–417. 10.1097/CEJ.0b013e3283639f7a 23817433 10.1097/CEJ.0b013e3283639f7a

[84] Rostami S, Kohan L, Mohammadianpanah M (2015) The LEP G-2548A gene polymorphism is associated with age at menarche and breast cancer susceptibility. Gene 557:154–157. 10.1016/j.gene.2014.12.021 25510398 10.1016/j.gene.2014.12.021

[85] Sangaramoorthy M, Koo J, John EM (2018) Intake of bean fiber, beans, and grains and reduced risk of hormone receptor-negative breast cancer: the San Francisco Bay Area Breast Cancer Study. Cancer Med 7:2131–2144. 10.1002/cam4.1423 29573201 10.1002/cam4.1423 PMC5943543

[86] Sepandi M, Akrami M, Tabatabaee H et al (2014) Breast cancer risk factors in women participating in a breast screening program: a study on 11,850 Iranian females. Asian Pac J Cancer Prev 15:8499–8502. 10.7314/APJCP.2014.15.19.8499 25339054 10.7314/apjcp.2014.15.19.8499

[87] Shirazi L, Almquist M, Borgquist S et al (2016) Serum vitamin D (25OHD3) levels and the risk of different subtypes of breast cancer: a nested case–control study. Breast 28:184–190. 10.1016/j.breast.2016.06.002 27326980 10.1016/j.breast.2016.06.002

[88] Silva AMC, Campos PHN, Mattos IE et al (2019) Environmental exposure to pesticides and breast cancer in a region of intensive agribusiness activity in Brazil: a case–control study. Int J Environ Res Public Health. 10.3390/ijerph16203951 31627286 10.3390/ijerph16203951 PMC6843507

[89] Sisti JS, Bernstein JL, Lynch CF et al (2015) Reproductive factors, tumor estrogen receptor status and contralateral breast cancer risk: results from the WECARE study. Springerplus 4:1–11. 10.1186/s40064-015-1642-y 26751177 10.1186/s40064-015-1642-y PMC4695460

[90] Sufian SN, Masroor I, Mirza W et al (2015) Evaluation of common risk factors for breast carcinoma in females: a hospital based study in Karachi, Pakistan. Asian Pac J Cancer Prev 16:6347–6352. 10.7314/APJCP.2015.16.15.6347 26434841 10.7314/apjcp.2015.16.15.6347

[91] Sun Y, Chen Q, Liu P et al (2021) Impact of traditional Chinese medicine constitution on breast cancer incidence: A case–control and cross-sectional study. Pharmacophore 12:46–56. 10.51847/uquyzry0b0

[92] Szkiela M, Kusideł E, Makowiec-Dabrowska˛ T, Kaleta D, (2020) Night shift work—a risk factor for breast cancer. Int J Environ Res Public Health. 10.3390/ijerph17020659 31968538 10.3390/ijerph17020659 PMC7013618

[93] Tan MM, Ho WK, Yoon SY et al (2018) A case-control study of breast cancer risk factors in 7663 women in Malaysia. PLoS ONE. 10.1371/journal.pone.0203469 30216346 10.1371/journal.pone.0203469 PMC6138391

[94] Tayour C, Ritz B, Langholz B et al (2019) A case–control study of breast cancer risk and ambient exposure to pesticides. Environ Epidemiol. 10.1097/EE9.0000000000000070 32166211 10.1097/EE9.0000000000000070 PMC7028467

[95] Thakur P, Seam RK, Gupta MK et al (2017) Breast cancer risk factor evaluation in a Western Himalayan state: a case–control study and comparison with the Western World. South Asian J Cancer 6:106–109. 10.4103/sajc.sajc_157_16 28975116 10.4103/sajc.sajc_157_16 PMC5615877

[96] Truong T, Liquet B, Menegaux F et al (2014) Breast cancer risk, nightwork, and circadian clock gene polymorphisms. Endocr Relat Cancer 21:629–638. 10.1530/ERC-14-0121 24919398 10.1530/ERC-14-0121

[97] Tumas N, Niclis C, Aballay LR et al (2014) Traditional dietary pattern of South America is linked to breast cancer: an ongoing case-control study in Argentina. Eur J Nutr 53:557–566. 10.1007/s00394-013-0564-0 23907208 10.1007/s00394-013-0564-0

[98] Veisy A, Lotfinejad S, Salehi K, Zhian F (2015) Risk of breast cancer in relation to reproductive factors in North-west of Iran, 2013–2014. Asian Pac J Cancer Prev 16:451–455. 10.7314/APJCP.2015.16.2.451 25684470 10.7314/apjcp.2015.16.2.451

[99] Wahidin M, Djuwita R, Adisasmita A (2018) Oral contraceptive and breast cancer risks: A case control study in six referral hospitals in Indonesia. Asian Pac J Cancer Prev 19:2199–2203. 10.22034/APJCP.2018.19.8.2199 30139225 10.22034/APJCP.2018.19.8.2199 PMC6171374

[100] Wang P, Ren FM, Lin Y et al (2015) Night-shift work, sleep duration, daytime napping, and breast cancer risk. Sleep Med 16:462–468. 10.1016/j.sleep.2014.11.017 25794454 10.1016/j.sleep.2014.11.017

[101] Wang S, Ogundiran T, Ademola A et al (2018) Development of a breast cancer risk prediction model for women in Nigeria. Cancer Epidemiol Biomarkers Prev 27:636–643. 10.1158/1055-9965.EPI-17-1128 29678902 10.1158/1055-9965.EPI-17-1128 PMC6086588

[102] Weiwei Z, Liping C, Dequan L (2014) Association between dietary intake of folate, Vitamin B6, B12 & MTHFR, MTR genotype and breast cancer risk. Pak J Med Sci 30:106–110. 10.12669/pjms.301.4189 24639841 10.12669/pjms.301.4189 PMC3955552

[103] Yaghjyan L, Colditz GA, Rosner B, Tamimi RM (2015) Mammographic breast density and breast cancer risk: interactions of percent density, absolute dense, and non-dense areas with breast cancer risk factors. Breast Cancer Res Treat 150:181–189. 10.1007/s10549-015-3286-6 25677739 10.1007/s10549-015-3286-6 PMC4372799

[104] Yang W, Shi Y, Ke X et al (2019) Long-term sleep habits and the risk of breast cancer among Chinese women: a case–control study. Eur J Cancer Prev 28:323–329. 10.1097/CEJ.0000000000000458 30188375 10.1097/CEJ.0000000000000458

[105] Zuraidah E, Agatha NFP, Edwar SQ, Handayani SI (2023) Correlation between age at first menarche and breast cancer in Dr. Cipto Mangunkusumo National General Hospital Jakarta in 2010–2014. Asian Pacific J Cancer Care 8:459–464. 10.31557/APJCC.2023.8.3.459

[106] McPherson K, Steel CM, Dixon JM (2000) Breast cancer-epidemiology, risk factors, and genetics. BMJ Br Med J 321:624–628 10977847 10.1136/bmj.321.7261.624 PMC1118507

[107] Macias H, Hinck L (2012) Mammary gland development. Wiley Interdiscip Rev Dev Biol 1:533–557. 10.1002/wdev.35 22844349 10.1002/wdev.35 PMC3404495

[108] Russo J, Russo IH (2004) Development of the human breast. Maturitas 49:2–15. 10.1016/j.maturitas.2004.04.011 15351091 10.1016/j.maturitas.2004.04.011

[109] Oh H, Bodelon C, Palakal M et al (2016) Ages at menarche- and menopause-related genetic variants in relation to terminal duct lobular unit involution in normal breast tissue. Breast Cancer Res Treat 158:341–350. 10.1007/s10549-016-3859-z 27342457 10.1007/s10549-016-3859-z PMC5144736

[110] Goldberg M, D’Aloisio AA, O’Brien KM et al (2020) Pubertal timing and breast cancer risk in the Sister Study cohort. Breast Cancer Res. 10.1186/s13058-020-01326-2 33109223 10.1186/s13058-020-01326-2 PMC7590599

[111] Lawson J, Field A, Champion S et al (1999) Low oestrogen receptor α expression in normal breast tissue underlies low breast cancer incidence in Japan. Lancet 354:1787–1788. 10.1016/S0140-6736(99)04936-3 10577642 10.1016/s0140-6736(99)04936-3

[112] Nagata C, Matsubara T, Fujita H et al (2005) Mammographic density and the risk of breast cancer in Japanese women. Br J Cancer 92:2102–2106. 10.1038/sj.bjc.6602643 15956963 10.1038/sj.bjc.6602643 PMC2361821

[113] Badr LK, Bourdeanu L, Alatrash M, Bekarian G (2018) Breast cancer risk factors: A cross- cultural comparison between the west and the east. Asian Pac J Cancer Prev 19:2109–2116. 10.22034/APJCP.2018.19.8.2109 30139209 10.22034/APJCP.2018.19.8.2109 PMC6171412

[114] Ziegler RG, Hoover RN, Pike MC et al (1993) Migration patterns and breast cancer risk in Asian-American women. J Natl Cancer Inst 85:1819–1827. 10.1093/jnci/85.22.1819 8230262 10.1093/jnci/85.22.1819

[115] van der Velden J, van Lindert ACM, Gimbrere CHF et al (1996) Epidemiologic data on vulvar cancer: comparison of hospital with population-based data. Gynecol Oncol 62:379–383. 10.1006/gyno.1996.0252 8812536 10.1006/gyno.1996.0252

[116] Mapel DW, Hunt WC, Utton R et al (1998) Idiopathic pulmonary fibrosis: survival in population based and hospital based cohorts. Thorax 53:469–476. 10.1136/thx.53.6.469 9713446 10.1136/thx.53.6.469 PMC1745251

[117] Giroud M, Lemesle M, Quantin C et al (1997) A hospital-based and a population-based stroke registry yield different results: the experience in Dijon, France. Neuroepidemiology 16:15–21. 10.1159/000109666 8994936 10.1159/000109666

[118] Ruano-Ravina A, Pérez-Ríos M, Miguel Barros-Dios J (2008) Population-based versus hospital-based controls: are they comparable? Gac Sanit 22:609–613. 10.1016/S0213-9111(08)75363-9 19080941 10.1016/s0213-9111(08)75363-9

[119] Wacholder S, Silverman DT, McLaughlin JK, Mandel JS (1992) Selection of controls in case-control studies: II. Types of controls. Am J Epidemiol 135:1029–1041. 10.1093/oxfordjournals.aje.a116397 1595689 10.1093/oxfordjournals.aje.a116397

[120] Eveleth PB (2017) Timing of Menarche: Secular Trend and Population Differences. In: Lancaster JB, Hamburg BA (eds) School-Age Pregnancy & Parenthood. Routledge, pp 39–52

[121] Sedgwick P (2014) Retrospective cohort studies: advantages and disadvantages. BMJ. 10.1136/bmj.g107225527114

[122] Yang PJ, Hou MF, Ou-Yang F et al (2022) Association of early-onset breast cancer with body mass index, menarche, and menopause in Taiwan. BMC Cancer. 10.1186/s12885-022-09361-2 35277131 10.1186/s12885-022-09361-2 PMC8917681

