# Supplementary material for: Early menarche and breast cancer risk: a systematic review and meta-analysis of 74 case–control studies
Source: Cancer Causes Control. 2026 Jan 17;37(2):32. doi: 10.1007/s10552-025-02096-y (PMC12812095; doi:10.1007/s10552-025-02096-y)
Supplement: Supplementary file 1 — Supplementary file1 (DOCX 48 KB) [file 10552_2025_2096_MOESM1_ESM.docx]

| Author | Year | Country | Region | Ethnicity | Breast cancer subtypes | Study design | Total sample size (Case/Control) | Source of control | Matching criteria | Menarche age (years old) | NOS Score |
| --- | --- | --- | --- | --- | --- | --- | --- | --- | --- | --- | --- |
| Alsolami et al.^32^ | 2019 | Saudi Arabia | West Asia | Arabic | N/A | Case-control | 214/218 | Hospital-based | Age | <10 | 6 |
| Amadou et al.^33^ | 2014 | Mexico | North America | Mexican | N/A | Case-control | 1000/1074 | Population-based | Age, healthcare system, region | <13 | 7 |
| Ambrosone et al.^34^ | 2015 | United States | North America | African | ER+, ER-, triple-negative | Case-control | 4400/17325 | Population-based | Age, race, area of residence | <13 | 6 |
| Amirzargar et al.^35^ | 2017 | Iran | West Asia | Iranian | N/A | Case-control | 182/158 | Hospital-based | Region | <12 | 7 |
| Andarieh et al.^36^ | 2019 | Iran | West Asia | Iranian | N/A | Case-control | 1177/1204 | Hospital-based | Age, area of residence | <12 | 7 |
| Arthur et al.^37^ | 2017 | United States | North America | American | N/A | Case-control nested within cohort | 526/526 | Hospital-based | Age at diagnosis of benign breast disease | <12 | 7 |
| Assi et al.^38^ | 2014 | United Kingdom | Europe | British | N/A | Case-control nested within cohort | 101/191 | Population-based | Age at recruitment, age at last mammogram prior to date of  diagnosis/pseudo-diagnosis | <13 | 7 |
| Babita et al.^39^ | 2014 | India | South Asia | Indian | N/A | Case-control | 122/120 | Hospital-based | Institute of recruitment | <13 | 5 |
| Balekouzou et al.^40^ | 2017 | Bangui, Central African Republic | Africa | African | N/A | Case-control | 174/348 | Hospital-based | Age | <12 | 7 |
| Beaber et al.^41^ | 2014 | United States | North America | Non-Hispanic White, African-American, Asian/Pacific Islander, Other | ER+, ER-, triple-negative, HER2-overexpressing | Case-control | 984/880 | Population-based | Age at recruitment | <12 | 7 |
| Berrandou et al.^42^ | 2019 | France | Europe | French | N/A | Case-control | 1111/1160 | Population-based | Age, area of residence | <13 | 6 |
| Bolognesi et al.^43^ | 2014 | Italy | Europe | Italian | N/A | Case-control | 184/266 | Hospital-based | N/A | <13 | 5 |
| Bravi et al.^44^ | 2018 | Italy | Europe | Italian | N/A | Case-control nested within cohort | 3223/9657 | Population-based | Age, year of enrolment | <12 | 8 |
| Chollet-Hinton et al.^45^ | 2016 | United States | North America | White, African-American, Other | Luminal, basal-like, HER2-overexpressing, unclassified (negative for all markers) | Case-control | 1033/870 | Population-based | Age, race | <13 | 6 |
| Clendenen et al.^46^ | 2015 | Sweden | Europe | Swedish | ER+, ER- | Case-control nested within prospective cohort | 713/1388 | Population-based | Age at enrolment, date of enrolment | <13 | 7 |
| Danjou et al.^47^ | 2019 | France | Europe | French | ER+, ER-, PR+, PR- | Case-control nested within cohort | 429/716 | Population-based | Age, area of residence, menopausal status, date of enrolment | <13 | 7 |
| Dianatinasab et al.^48^ | 2017 | Iran | West Asia | Iranian | N/A | Case-control | 506/519 | Hospital-based | Age | <12 | 6 |
| Dydjow-Bendek & Zagoźdźon^49^ | 2020 | Poland | Europe | Polish | N/A | Case-control | 201/201 | Population-based | Age, socio-economic status | <13 | 8 |
| Elkum et al.^50^ | 2014 | Saudi Arabia | West Asia | Arab | N/A | Case-control | 534/638 | Hospital-based | Unmatched | <13 | 5 |
| Ellingjord-Dale et al.^51^ | 2017 | Norway | Europe | Norwegian | Luminal A, luminal B, HER2-positive, triple-negative | Case-control nested within cohort | 5352/25932 | Population-based | Area of residence | <13 | 7 |
| Eng et al.^52^ | 2014 | United Kingdom | Europe | White, Other | N/A | Case-control | 414/685 | Hospital-based | N/A | <13 | 5 |
| Franke et al.^53^ | 2021 | United States | North America | Japanese America, White, Native Hawaiian | N/A | Case-control nested within cohort | 124/125 | Population-based | Birth year, race | <13 | 7 |
| Gago-Dominguez et al.^54^ | 2020 | Spain | Europe | Spanish | Luminal A, luminal B, HER2-positive, triple-negative | Case-control | 300/372 | Population-based | Area of residence | <13 | 8 |
| Ge et al.^55^ | 2015 | Germany | Europe | German | ER+, ER-, PR+, PR- | Case-control | 2887/5512 | Population-based | Birth year, study region | <12 | 7 |
| Giudici et al.^56^ | 2017 | Italy | Europe | Italian White | Luminal A, luminal B | Case-control | 281/573 | Population-based | Age | <13 | 7 |
| Glass et al.^57^ | 2015 | Australia | Oceania | Australian | ER+, ER- | Case-control | 1202/1785 | Population-based | Age | <13 | 6 |
| Goldberg et al.^58^ | 2017 | Canada | North America | French, English, Jewish, Italian | ER+, ER-, PR+, PR- | Case-control | 673/585 | Population-based | Age | <13 | 7 |
| Gravena et al.^59^ | 2018 | Brazil | South America | Brazilian | ER, PR, HER2, triple-negative | Case-control | 100/400 | Hospital-based | Age | <13 | 7 |
| Heikkinen et al.^60^ | 2015 | Finland | Europe | Finnish | N/A | Case-control | 5897/19544 | Population-based | Birth year | <13 | 5 |
| Hosseinzadeh et al.^61^ | 2014 | Iran | West Asia | Iranian | N/A | Case-control | 140/280 | Hospital-based | Age | <13 | 7 |
| Hudson et al.^62^ | 2018 | United Kingdom | Europe | White, Non-white | N/A | Case-control | 294/503 | Population-based | N/A | <13 | 5 |
| Hurley et al.^63^ | 2018 | United States | North America | White, Black, Hispanic, Asian/Pacific Islander, Other | ER+, ER-, PR+, PR- | Case-control nested within cohort | 893/847 | Population-based | Age, race, area of residence | <12 | 6 |
| Kawai et al.^64^ | 2014 | United States | North America | Non-Hispanic white, African American, Asian/Pacific Islander, Hispanic white | ER+, triple-negative | Case-control | 959/936 | Population-based | Age | <12 | 6 |
| Keller et al.^65^ | 2015 | United States | North America | Caucasian, African-American, Asian, Other | N/A | Case-control | 106/318 | Hospital-based | Age | <12 | 5 |
| Kim et al.^66^ | 2016 | United States | North America | White, Black, Other | N/A | Case-control | 1443/1462 | Population-based | Age | <13 | 7 |
| Krishnan et al.^67^ | 2016 | Australia | Oceania | Anglo Saxon, Italian, Greek | ER, PR, HER2 | Case-control nested within prospective cohort | 390/1146 | Population-based | Birth year, year of enrolment, country of origin | <13 | 6 |
| Laamiri et al.^68^ | 2016 | Morocco | Africa | Moroccan | N/A | Case-control | 124/148 | Hospital-based | N/A | <12 | 6 |
| Liu et al.^69^ | 2017 | China | East Asia | Chinese Han | Luminal A, luminal B, HER2-positive, triple-negative | Case-control | 1454/1462 | Hospital-based | Age, hospital of recruitment | <12 | 6 |
| Ma et al.^70^ | 2017 | United States | North America | White, African-American | Luminal A, luminal B, HER2-positive, triple-negative | Case-control | 2658/2448 | Population-based | Age, race, area of residence | <13 | 8 |
| Mohite et al.^71^ | 2015 | India | South Asia | Indian | N/A | Case-control | 217/217 | Hospital-based | Age, religion, area of residence | <13 | 6 |
| Mojahed et al.^72^ | 2020 | Iran | West Asia | Iranian | ER, PR, HER2 | Case-control | 36/36 | Hospital-based | N/A | <13 | 6 |
| Moore et al.^73^ | 2021 | United States | North America | Non-Hispanic white, Other | ER+ | Case-control nested within prospective cohort | 770/772 | Population-based | Birth date, race/ethnicity | <12 | 7 |
| Moradzadeh et al.^74^ | 2019 | Iran | West Asia | Iranian | N/A | Case-control | 880/998 | Hospital-based | Age, area of residence | <13 | 7 |
| Nagrani et al.^75^ | 2015 | India | South Asia | Indian | N/A | Case-control | 1637/1515 | Hospital-based | Age, area of residence, time of enrolment | <13 | 6 |
| Nishino et al.^76^ | 2014 | Japan | East Asia | Japanese | ER+/PR+, ER+/PR-, ER-/PR+, ER-/PR- | Case-control | 1263/3160 | Hospital-based | N/A | <13 | 5 |
| O’Brien et al.^77^ | 2015 | United States | North America | Non-Hispanic white, non-Hispanic black, Hispanic, Other | N/A | Sister-matched case-control | 1406/1647 | Population-based | Full sister | <12 | 6 |
| Pavanello et al.^78^ | 2018 | Italy | Europe | Italian | N/A | Case-control | 113/214 | Population-based | N/A | <13 | 5 |
| Pimhanam et al.^79^ | 2014 | Thailand | Southeast Asia | Thai | N/A | Case-control | 444/444 | Hospital-based | Age | <12 | 7 |
| Playdon et al.^80^ | 2017 | United States | North America | Non-Hispanic white, Other | ER+, ER-, PR- | Case-control nested within prospective cohort | 100/100 | Population-based | Age | <12 | 6 |
| Rashid et al.^81^ | 2015 | Pakistan | South Asia | Pakistani | ER, PR, HER2 | Case-control | 448/989 | Hospital-based | N/A | <13 | 5 |
| Ronco et al.^82^ | 2016 | Uruguay | South America | Uruguayans | N/A | Case-control | 572/889 | Hospital-based | Age, area of residence | <13 | 7 |
| Rosato et al.^83^ | 2014 | Italy | Europe | Italian | ER+, ER-, PR+, PR- | Case-control | 1075/1474 | Hospital-based | N/A | <13 | 5 |
| Rostami et al.^84^ | 2014 | Iran | West Asia | Iranian | N/A | Case-control | 203/171 | Hospital-based | Age, sex | <13 | 6 |
| Sangaramoorthy et al.^85^ | 2018 | United States | North America | Foreign-born Hispanic, U.S.-born Hispanic, African American, Non- Hispanic White | ER+, PR+, ER-/PR- | Case-control | 2135/2571 | Population-based | Age, race | <12 | 6 |
| Sepandi et al.^86^ | 2014 | Iran | West Asia | Iranian | N/A | Case-control | 197/11653 | Population-based | N/A | <12 | 7 |
| Shirazi et al.^87^ | 2016 | Sweden | Europe | Swedish | ER+, ER-, PR+, PR- | Case-control nested within cohort | 764/764 | Population-based | Age at enrolment, menopausal status, date of enrolment | <12 | 8 |
| Silva et al.^88^ | 2019 | Brazil | South America | East Asian, Black, White, Brown, Others | N/A | Case-control | 85/264 | Hospital-based | N/A | <13 | 5 |
| Sisti et al.^89^ | 2015 | United States | North America | American | ER+, ER-, PR+, PR- | Case-control | 1515/2204 | Population-based | Birth year, diagnosis year, cancer registry region, race/ethnicity | <13 | 5 |
| Sufian et al.^90^ | 2015 | Pakistan | South Asia | Pakistani | N/A | Case-control | 106/108 | Hospital-based | Age | <13 | 5 |
| Sun et al.^91^ | 2021 | Hong Kong, China | East Asia | Chinese | Luminal A, luminal B, HER2-overexpression, triple-negative | Cross-sectional, case-control | 305/303 | Population-based | N/A | <12 | 6 |
| Szkiela et al.^92^ | 2020 | Poland | Europe | Polish | N/A | Case-control | 473/493 | Hospital-based | Age, education | <13 | 5 |
| Tan et al.^93^ | 2018 | Malaysia | Southeast Asia | Chinese, Indian, Malay, Other | N/A | Case-control | 2892/3940 | Hospital-based | Unmatched | <13 | 5 |
| Tayour et al.^94^ | 2019 | United States | North America | Non-Hispanic  white | N/A | Case-control | 155/150 | Population-based | Postmenopausal, area of residence | <13 | 6 |
| Thakur et al.^95^ | 2020 | India | South Asia | Indian | N/A | Case-control | 377/346 | Hospital-based | Unmatched | <12 | 5 |
| Truong et al.^96^ | 2014 | France | Europe | French | ER+/PR+, ER+/PR-, ER-/PR+, ER-/PR- | Case-control | 955/958 | Population-based | Age, area of study | <13 | 7 |
| Tumas et al.^97^ | 2014 | Argentina | South America | Argentinean | N/A | Case-control | 75/180 | Population-based | Age, sex, area of residence | <12 | 7 |
| Veisy et al.^98^ | 2015 | Iran | West Asia | Iranian | N/A | Case-control | 224/234 | Population-based | Age, socio-economic status, weight, parity | <13 | 7 |
| Wahidin et al.^99^ | 2018 | Indonesia | Southeast Asia | Indonesian | N/A | Case-control | 381/381 | Hospital-based | N/A | <13 | 6 |
| Wang et al.^100^ | 2018 | Ibadan, Nigeria | Africa | Nigerian | N/A | Case-control | 1811/2225 | Population-based | N/A | <13 | 5 |
| Wang et al.^101^ | 2014 | China | East Asia | Chinese | ER, PR, HER2 | Case-control | 705/737 | Hospital-based | Age | <13 | 5 |
| Weiwei et al.^102^ | 2014 | China | East Asia | Chinese | N/A | Case-control | 295/306 | Hospital-based | N/A | <13 | 4 |
| Yaghjyan et al.^103^ | 2015 | United States | North America | American | N/A | Case-control nested within cohort | 1044/1794 | Hospital-based | Age, menopausal status | <12 | 7 |
| Yang et al.^104^ | 2018 | China | East Asia | Chinese | ER+, ER- | Case-control | 401/401 | Population-based | Birth year, sex, area of residence | <12 | 7 |
| Zuraidah et al.^105^ | 2023 | Indonesia | Southeast Asia | Indonesian | N/A | Cross-sectional | 86/12 | Hospital-based | N/A | <13 | N/A |

**Supplementary Material 1.** Characteristics of the observational studies included in the meta-analysis on the association between age of menarche and female breast cancer risk.

NOS: Newcastle-Ottawa Scale; N/A: not available; ER: oestrogen receptor; PR: progesterone receptor; HER2: human epidermal growth factor receptor 2
